# Modulation of virtual arm trajectories via microstimulation in a spiking model of sensorimotor cortex

**DOI:** 10.1186/1471-2202-15-S1-P106

**Published:** 2014-07-21

**Authors:** Salvador Dura-Bernal, Kan Li, Austin J Brockmeier, Cliff C Kerr, Samuel A Neymotin, Jose C Principe, Joseph T Francis, William W Lytton

**Affiliations:** 1 Department of Physiology and Pharmacology, SUNY Downstate, Brooklyn, NY 11203, USA; 2 Department of Electrical and Computer Engineering, University of Florida, Gainesville, FL 32611, USA

## 

Electrical microstimulation can be used to drive neural responses to match meaningful spiking patterns corresponding to natural sensory stimuli or motor behaviors. Optimizing microstimulation sequences requires repeatedly stimulating the neural system to obtain sufficient probing data to construct an inverse model. This is challenging in the real brain where probing time may be limited and plasticity may be induced. Biologically realistic models allow the system to be repeatedly probed and reset, providing a unique test bed for understanding the dynamic interaction between ongoing neural activity and artificially applied stimulation. Here, we employ a biomimetic spiking model (BMM) of sensorimotor cortex which controls a realistic virtual musculoskeletal arm that performs reaching movements [1].

**Figure 1:**
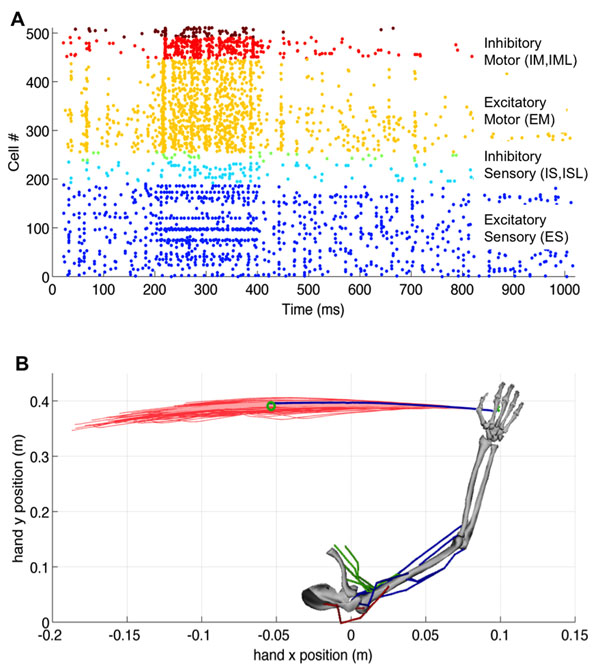
**A.** Raster plot of a multiple-neuron microstimulation probing sequence. **B.** Virtual arm with superimposed hand trajectories for single-neuron (light red) vs. multiple-neuron (dark red) microstimulation probing sequences (original trajectory in blue; target in green).

After training the BMM and virtual arm to reach a target, a set of 1536 microstimulation probing sequences were applied to the target sensory population. The output motor population activity and the virtual arm trajectories were recorded (Figure 1). Microstimulation was applied to each target neuron individually as well as to random subsets of multiple neurons simultaneously. Other parameters, such as the start time, duration, and amplitude of the stimulation, were also examined. Results demonstrated that microstimulation can differentially modulate the motor population which excites the arm muscles, and consequently, the hand trajectories. The results of the probing dataset will be used to build an inverse model of the neural system, which will be able to generate microstimulation patterns [2] to repair simulated lesions in the model.

This work demonstrates the advantages of employing in silico brain simulations and realistic limb models as a test bed for microstimlation-based neural controllers. The proposed system, which has been previously interfaced with a neural data acquisition system and a robotic arm in real time [1], paves the way for the faster development of neuroprosthetics for the dynamic repair of damaged motor neural systems.
